# Design, Synthesis and Pharmacological Evaluation of Novel Vanadium-Containing Complexes as Antidiabetic Agents

**DOI:** 10.1371/journal.pone.0100386

**Published:** 2014-07-24

**Authors:** Elena V. Fedorova, Anna V. Buryakina, Alexey V. Zakharov, Dmitry A. Filimonov, Alexey A. Lagunin, Vladimir V. Poroikov

**Affiliations:** 1 Saint-Petersburg State Chemical Pharmaceutical Academy, Ministry of Healthcare and Social Development of Russian Federation, Saint-Petersburg, Russian Federation; 2 National Cancer Institute, National Institutes of Health, Frederick, Maryland, United States of America; 3 Orekhovich Institute of Biomedical Chemistry of Russian Academy of Medical Sciences, Moscow, Russian Federation; National Cancer Institute at Frederick, United States of America

## Abstract

Based on the data about structure and antidiabetic activity of twenty seven vanadium and zinc coordination complexes collected from literature we developed QSAR models using the GUSAR program. These QSAR models were applied to 10 novel vanadium coordination complexes designed *in silico* in order to predict their hypoglycemic action. The five most promising substances with predicted potent hypoglycemic action were selected for chemical synthesis and pharmacological evaluation. The selected coordination vanadium complexes were synthesized and tested *in vitro* and *in vivo* for their hypoglycemic activities and acute rat toxicity. Estimation of acute rat toxicity of these five vanadium complexes was performed using a freely available web-resource (http://way2drug.com/GUSAR/acutoxpredict.html). It has shown that the selected compounds belong to the class of moderate toxic pharmaceutical agents, according to the scale of Hodge and Sterner. Comparison with the predicted data has demonstrated a reasonable correspondence between the experimental and predicted values of hypoglycemic activity and toxicity. Bis{tert-butyl[amino(imino)methyl]carbamato}oxovanadium (IV) and sodium(2,2′-Bipyridyl)oxo-diperoxovanadate(V) octahydrate were identified as the most potent hypoglycemic agents among the synthesized compounds.

## Introduction

Vanadium is a biologically important element involved in numerous physiological processes. The presence of transition valence determines wide distribution of vanadium compounds in nature that are found only in chemically combined form. Vanadium compounds, in particular organic derivatives, are effective oral insulinomimetics, which inhibit lipolysis, decrease blood glucose levels (BGL) in animals and in clinical trials, and stimulate insulin secretion in experimental models of Diabetes Mellitus (DM) [1]–[5]. Although mechanisms of the insulin mimetic effect of vanadium complexes still have to be clarified, their ability to sensitize peripheral tissues to insulin and to reduce insulin resistance attracts significant attention in the context of their potential use for the treatment of Diabetes Type 1 (DM1), Diabetes Type 2 (DM2) and obesity [6], [7].

According to the Integrity database (http://integrity.thomson-pharma.com), vanadium compounds have been developed as drugs for treatment of Diabetes, Hypertension, Ischemia and Myocardial Stroke.

The major problem limiting further clinical applications of inorganic vanadium compounds is a low oral absorption (1–10%). However, it was shown that the oral bioavailability of vanadium complexes with organic ligands exceeded 20–40%, which can be considered as acceptable level of bioavailability [8].

Currently QSAR modeling is widely applied in rational drug design [9]. Despite the many examples of successful application of QSAR for finding and optimization of new lead compounds, only few QSAR studies on biological activities of vanadium complexes have been reported (see, e.g., [10]). Limited number of QSAR applications to the metal-complexes may be explained by the difficulties with the development of chemical descriptors, which reflect well the quantitative structure-activity relationships [11]. Recently a novel QSAR approach based on Quantitative Neighborhoods of Atoms (QNA) descriptors and self-consisted regression has been proposed; and its superiority in comparison with many other popular methods was shown [12]. This approach was realized in GUSAR program: General Unrestricted Structure-Activity Relationships. Since GUSAR was successfully applied for compounds from different chemical series and various activity/property endpoints [12], it was interesting to investigate if it would provide the accurate prediction results for vanadium complexes with antidiabetic activity. Moreover, GUSAR can be used not only as a tool for creation of QSAR models for the series of compounds with known activity values but also for estimating the acute rats toxicity (LD_50_ values) for compounds, according to different route of administration (oral, intravenous, intraperitoneal and subcutaneous) [13].

In this study we apply the computer program GUSAR to analyze the structure-activity relationships of vanadium complexes with antidiabetic action, predict antidiabetic activity and toxicity of novel designed compounds, select the most promising of them for synthesis and biological testing, and verify the computational predictions by the experiment.

## Results

### Quantitative structure-activity relationships (QSAR)

We collected information about structures of the twenty seven zinc and vanadium complexes with (O4), (N2O2), (N2S2), (O2S2), and (S4) coordination modes and their hypoglycemic activity from the available sources (File S1). They have been reported to display considerable insulinomimetic effects in *in vitro* experiments on enzyme inhibitions and blood glucose-lowering effects. The hypoglycemic activity was presented as 50 percent inhibitory concentration (IC_50_). Both zinc and vanadium complexes were used for QSAR analysis. The collected data was randomly divided twenty times onto the training and test sets with a ratio of 70% and 30%, respectively. Here we call this multiple splitting procedure a leave-30%-out cross-validation (L30%CV). This procedure is similar to n-fold external validation, but there is only one difference. During the n-fold validation each compound may be used as a test compound only once, but during the multiple splitting validation each compound may be used as a test compound several times, which depends from splitting. The training sets were used for creation of QSAR models and test sets for the assessment of external predictive accuracy. In addition to L30%CV we have used the procedure of selection of the test set compounds taking into account the activity distribution (Act.Dis.) using the same proportion as mentioned above: 70% for the training set and 30% for the test set. QSAR models were developed using GUSAR program (version 2011), which based on Multilevel and Quantitative Neighborhoods of Atoms (MNA, QNA) descriptors [14], [15] and self-consistent regression (SCR) algorithm [12], [13].

Two hundred QNA and MNA based models were generated for each training set. Models that satisfied the following criteria were considered as acceptable for prediction: I) R^2^_test_>0.5, II) Q^2^>0.5. As a result, 14 acceptable models were selected. Characteristics of the obtained models as well as the results of external predictivity of each model calculated by multiple splitting procedure (L30%CV) and by activity distribution procedure (L30%Act.Dis) are presented in Table 1. According to the results described in Table 1 the multiple splitting procedure provides more reliable assessment of the predictivity of models compared to the activity distribution procedure, which is overoptimistic. Thus, selection of models had been done by L30%CV. Only QNA based models were satisfied the described criteria. Thus, QNA descriptors are more suitable for modeling of complex compounds compared to MNA descriptors.

**Table 1:** Characteristics of QSAR models.

Model Number	Descriptors	Compound’s Number	R^2^	Q^2^	Fisher	SD	Number of Variables	R^2^ test,L30%Out	R^2^ test,L30%Act.Dis
Model 1	QNA,V	27	0.782	0.576	12.766	0.186	6	0.524	0.604
Model 2	QNA,Lip	27	0.715	0.561	7.452	0.209	7	0.711	0.801
Model 3	QNA,Lip	27	0.711	0.548	7.363	0.21	7	0.588	0.658
Model 4	QNA,Lip	27	0.782	0.612	9.198	0.198	8	0.624	0.724
Model 5	QNA,Lip	27	0.797	0.586	11.574	0.186	7	0.543	0.623
Model 6	QNA	27	0.725	0.515	8.039	0.21	7	0.635	0.705
Model 7	QNA	27	0.696	0.528	8.504	0.215	6	0.622	0.712
Model 8	QNA	27	0.799	0.682	13.989	0.177	6	0.582	0.672
Model 9	QNA,Lip	27	0.808	0.628	12.378	0.181	7	0.677	0.747
Model 10	QNA,Lip	27	0.748	0.577	7.42	0.204	8	0.627	0.727
Model 11	QNA,Lip	27	0.809	0.628	12.383	0.181	7	0.646	0.726
Model 12	QNA	27	0.74	0.527	8.618	0.206	7	0.62	0.72
Model 13	QNA	27	0.701	0.501	6.14	0.221	8	0.827	0.907
Model 14	QNA	27	0.774	0.588	8.766	0.201	8	0.501	0.591

V – Volume descriptor, Lip – logP descriptor, L30% Out – leave-30%-out cross-validation procedure, L30%Act.Dis – leave-30%-out splitting procedure by activity distribution.

Selected models were used for consensus prediction of 10 novel vanadium coordination complexes designed *in silico*, to estimate their hypoglycemic activity. Prediction results obtained using the consensus model for 10 novel compounds are presented in Table 2.

**Table 2:** Prediction results achieved by consensus model for 10 novel compounds.

Compound	Organic ligands	IC_50_ Predicted	Name of the ligand
1	C_10_N_2_	0.5	Bipyridine
2	C_5_H_7_O_2_	0.53	Acetyl acetone
3	C_6_H_12_O_2_N_3_	0.75	Tert-butyl[amino(imino)methyl]carbamate
4	C_10_H_11_NO_3_	0.87	2-(*N*-salicylidene)aminopropionate
5	C_4_H_6_O_6_	0.93	Tartaric acid
VOSO_4_ as reference compound inorganicvanadium salt IC_50_ = 1.00±0.09	none	1.00	none
6	C_8_H_4_Cl_2_N_2_OS	1.12	1,3,4-oxadiazole-2-thiol, 5-(2,4-dichlorophenyl)-
7	C_8_H_5_ClN_2_OS	1.13	1,3,4-oxadiazole-2-thiol, 5-(4-chlorophenyl)-
8	C_8_H_6_N_2_O_2_S	1.16	3-(5-mercapto-1,3,4-oxadiazol-2-yl)-
9	C_8_H_5_BrN_2_OS	1.16	5-(3-bromophenyl)-1,3,4-oxadiazole-2-thiol
10	C_8_H_6_N_2_OS	1.21	1,3,4-oxadiazole-2-thiol, 5-phenyl-

Five most active compounds according to the prediction results described in Table 2 were selected for synthesis and hypoglycemic activity testing.

### Synthesis

Based on prediction, we have selected for future synthesis five vanadium complexes that are expected to be more effective in lowering the glucose concentration in blood serum than VOSO_4_ that is usually used as a reference substance [16]. Among the selected structures the vanadium complexes in various oxidation states (both neutral and anionic forms) were found, which contain a variety of coordination modes: oxocomplexes **II**, **III** and **IV** of vanadium(IV) – the general formula VOL_2_; oxocomplexes **V** of vanadium(V) with tridentate ligand, the general formula [VO(H_2_O)(L)]; and oxodiperoxo complexes **I** of vanadium(V) - the general formula [VO(O_2_)_ 2_(L)_2_]^n-^, where n = 1.

**Figure 1:**
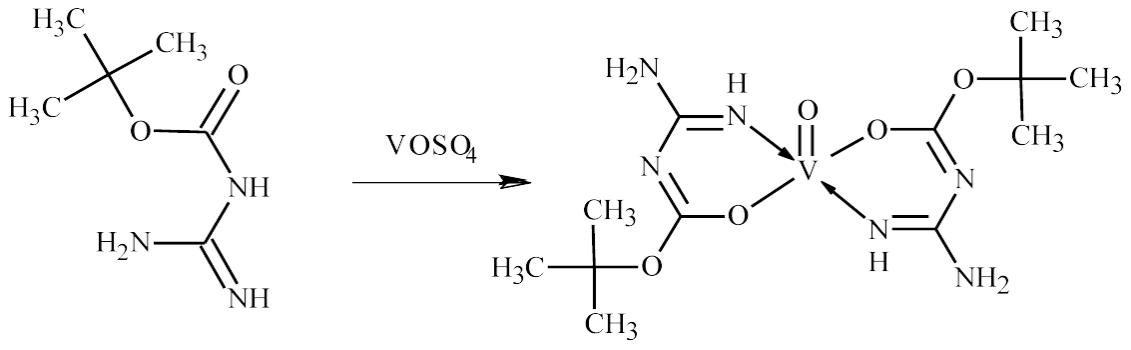
Synthesis of oxovanadium complexes with tert-butyl [amino(imino)methyl] carbamate ligand.

**Figure 2:**
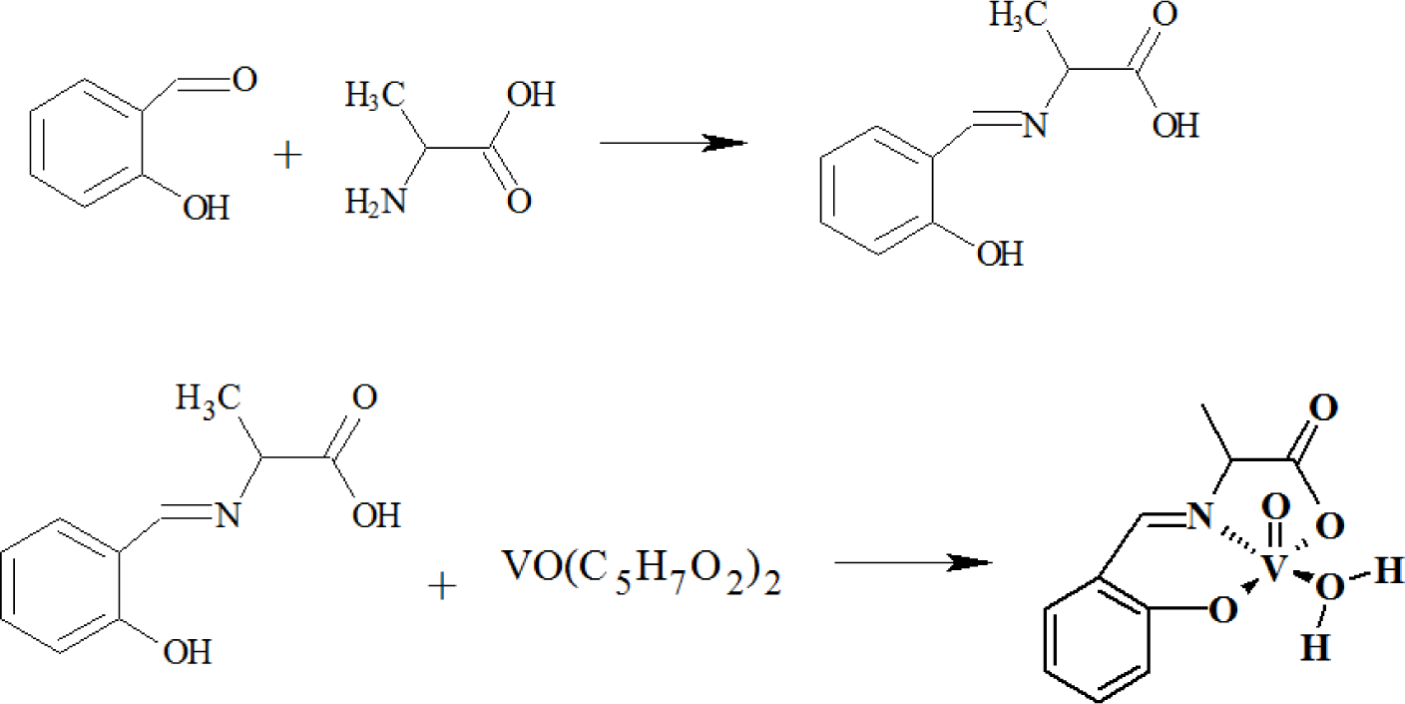
Synthesis of 2-(*N*-salicylidene)aminopropionate and their vanadium oxocomplex.

Complex **I** was formed by the interaction of sodium metavanadate with bidentate chelating ligand 2,2′-Bipyridine (C_10_H_8_N_2_) and hydrogen peroxide:
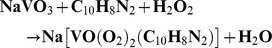



Formation of complex **II** is a two-step reaction. First, V_2_O_5_ is reduced to vanadyl ion [VO]^2+^ which is found as vanadyl sulphate ([V(O)(H_2_O)_4_]SO_4_):
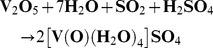



Then, vanadyl sulphate reacts with acetyl acetone in basic medium:
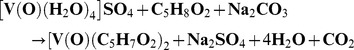



and the overall reaction is:
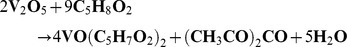



Complex **IV** was prepared by reacting of tartaric acid with sodium vanadium oxide (V) under heating and further neutralization with potassium hydroxide solution:
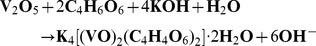



Complex **III** and complex **V** were formed according to Fig. 1 and Fig. 2 respectively.

**Figure 3:**
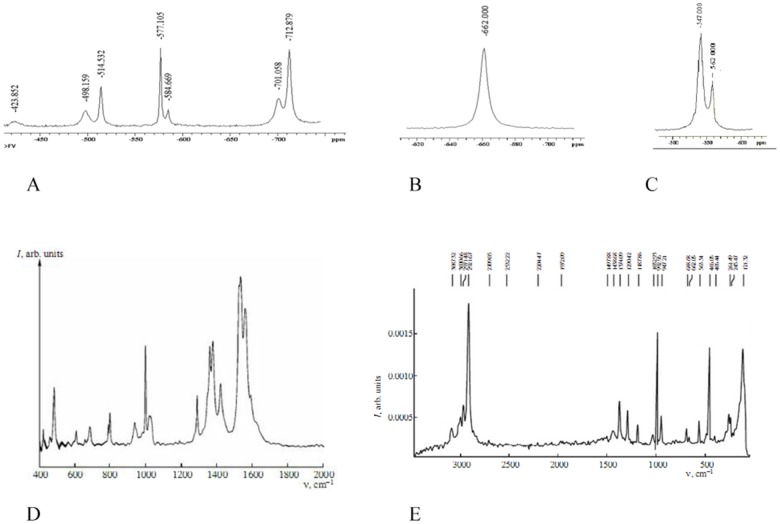
51V-NMR spectra for vanadium (V). The wide spectral dispertion of the signals as characteristic of vanadium NMR spectra (**A**) complex **I** (**B**); complex **V** (**C**). IR (**D**) and Raman (**E**) spectra for vanadium complex **II**.

**Figure 4:**
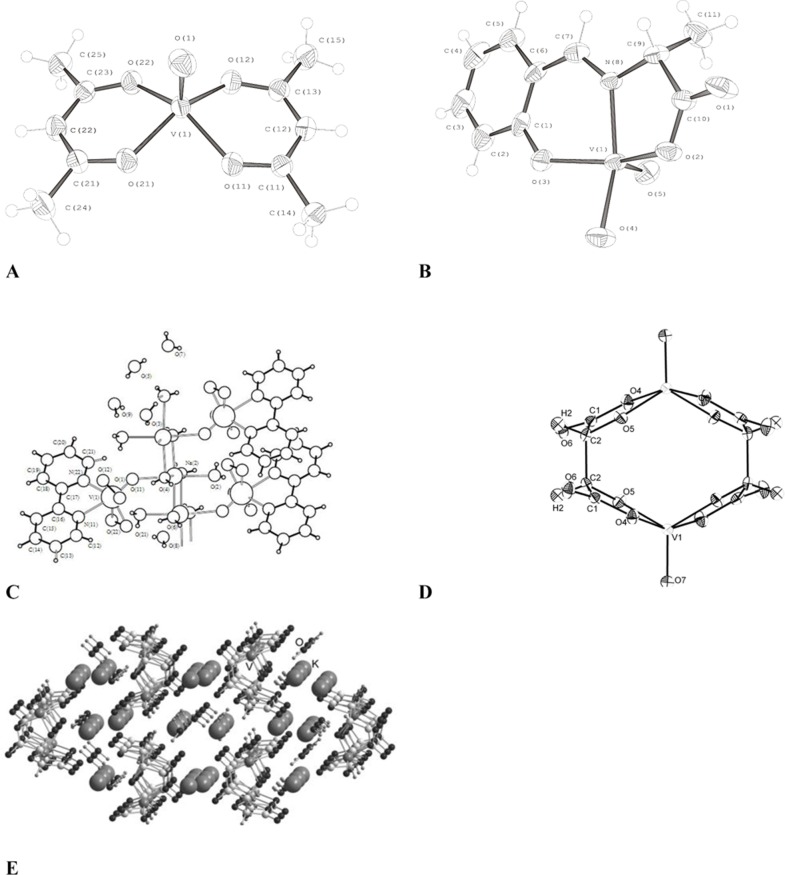
Molecular structure and the atomic numbering for complex I(C), complex II(A), complex IV(D, E) and complex V(B). The ellipsoids of thermal vibrations are shown at the 50% probability level.

The information about structure of complexes is summarized in **Table 3**. The chemical structures of these compounds were characterized by ^1^H-, ^51^V-NMR spectra for vanadium(V) complexes (**Fig. 3A–C**), IR and Raman spectra for vanadium(IV) and (V) complexes (**Fig. 3D, E**). The crystal structures of the vanadium complexes **I**, **II**, **IV** and **V** were confirmed by X-ray diffraction analysis (**Fig. 4** **A–E**).

**Table 3:** Structures of synthesized vanadium complexes I–V.

Complexes	Formula	Name	Abbreviation in article
Complex I (anionic)	Na[VO(O_2_)_2_(C_10_H_8_N_2_)]·8H_2_O	Sodium(2,2′-Bipyridyl) oxo-diperoxovanadate(V) octahydrate	2,2′-Bipy
Complex II (neutral)	[VO(C_5_H_7_O_2_)_2_]	Bis(acetylacetonato)oxo-vanadium(IV)	acac
Complex III (neutral)	[VO(C_6_H_12_O_2_N_3_)_2_]	Bis{tert-butyl[amino(imino) methyl] carbamato} oxovanadium (IV)	bamc
Complex IV (anionic)	K_4_[V_2_O_2_(C_4_H_4_O_6_)_2_]·2H_2_O	Potassium bis((m2-d,d-tartrato-O,O′,O′′,O′′′)-oxo-vanadium(IV)) dihydrate	tartrate
Complex V (neutral)	[VO(C_10_H_11_NO_3_)(H_2_O)]	(S)-[2-(N-salicylidene) amino-propionate] oxovanadium(IV) monohydrate	salen

## Discussion

The developed QSAR models were applied to 10 novel vanadium coordination complexes designed *in silico* to predict their hypoglycemic activity. The organic molecules with donor atoms, preferably N and O, were used as ligands since the cations of vanadium (IV) and vanadium (V) form a stronger bond with oxygen than to nitrogen.

It is well known that both V(IV) and V(V) complexes show insulin mimetic effects [6], however it is generally considered that the most effective insulin-mimetic vanadium complexes are neutral V(IV) compounds of the general formula VO[L_2_]^0^. Several such VOL_2_ type complexes with hypoglycemic activity have already been patented. The bis(maltolato)oxovanadium(IV) BMOV was introduced into clinical tests several years ago [17]. Also, an ethyl-derivative of BMOV has been recently entered into clinical trials [18].

On the other hand, vanadium complexes with oxidation state +V electronically and structurally imitate phosphate. Recent studies have also demonstrated that some of pentavalent vanadium complexes with organic ligands are much less toxic than caffeine (LD_50_ is 192 mg/kg) and oxocompex - BMOV (LD_50_ is 220 mg/kg) and, in addition, they are more soluble and bioavailable [17].

Therefore, predictions were made for various vanadium structures such as well known VOL_2_ type and for the vanadium (V) complexes that, in our opinion, could be also very efficient as insulin mimetic agents. All compounds match to the applicability domain of the obtained QSAR models. Five most promising substances with predicted potent hypoglycemic action were selected for chemical synthesis.

Various approaches were applied for the synthesis of vanadium complexes **I**–**V** (**see Methods**), using as a starting material both inorganic vanadium(IV) and vanadium(V) compounds.

To analyze the behavior in physiological solutions and to determine the structure of vanadium complexes we used various physico-chemical methods (IR, NMR, X-Ray). Vanadium - 51 (nuclear spin = 7/2) is about 40% as sensitive as protons toward NMR observation, and therefore spectra are generally easily obtained (Fig. 3A). Method ^51^V-NMR accurately recognizes the specific behavior of compounds V (V) in solutions. During registration of ^51^V-NMR spectra as the external standard VOCl_3_ was used.

**Figure 5:**
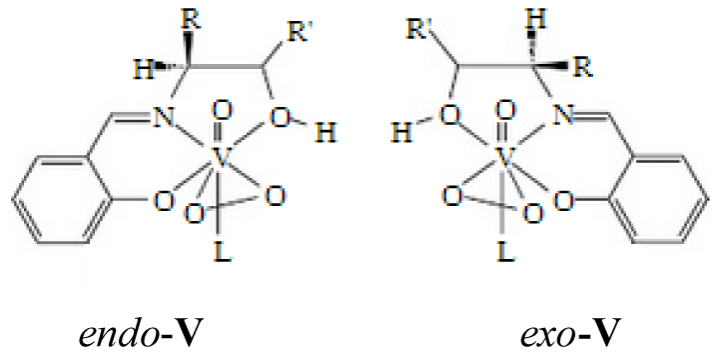
Structures of two diastereomeric forms of complex V, R = CH_3_, R′ = O.

We used the method of ^51^V-NMR spectroscopy for the study of diamagnetic vanadium complexes **I** and **V**. The ^51^V-NMR spectrum of complex **I** is represented by a singlet at –662 ppm (Fig. 3B) typical of oxodiperoxovanadium complexes. The ^51^V- NMR spectra of complex **V** shows two signals in the range from –540 to –565 ppm (Fig. 3C) corresponding to oxovanadium complexes. The presence of two signals in the spectra of complex **V** can be evidence of the existence of two diastereomeric *endo* and *exo* forms of that complex (Fig. 5).

Infrared spectroscopy is used mainly to prove the presence of functional groups in the molecule. Thus, the stretching vibrations of the V = O in oxocomplexes of tetravalent vanadium is usually found at higher frequencies compared to the oxocomplexes of pentavalent vanadium. As an example in the IR spectra of complexes **II** the strongest bands at 980 cm^−1^ correspond to the V = O stretching vibrations (Fig. 3D). In the Raman spectrum of complex **II**, the most intense lines observed at frequencies of 993 and 466 cm^–1^ correspond to the stretching vibrations of the V = O bonds and to the totally symmetric vibrations of the V–O bonds, respectively. The medium-intensity line at 610 cm^–1^ and an intense line at 486 cm^–1^ are associated with the two other among the three remaining of VO_4_ vibrations (Fig. 3E).

The most intense bands in the IR spectrum of the synthesized complex **I** correspond to vibrations of crystal water molecules (3450–3200 cm^–1^) and to the stretching vibrations of the oxodiperoxovanadium moiety: the band at 926 cm^–1^ is due to the V = O vibrations, the bands at 879 and 858 cm^–1^ correspond to O–O bond vibrations. The broad band at 586 cm^–1^ and the medium-intensity band at 624 cm^–1^ are produced by the symmetric and antisymmetric vibrations of the VO_2_ fragment, respectively. The positions of these bands coincide with published data to within 4 cm^–1^ [19]. The band at 766 cm^–1^, which virtually retains its position in the spectrum of crystalline *2, 2′-Bipy*, and a number of weak bands in the region of 1200–950 cm^–1^ can be attributed to ligand vibrations. The positions and intensities of most of the *2, 2*′*-Bipy* bands in the IR spectrum of complex **I**, primarily in the region of 1700–1300 cm^–1^, are markedly different from those observed for ligand-free form *2, 2*′*-Bipy* (**see abbreviation in** **Table 3**), which is due to the conformational change that takes place in the *2, 2*′*-Bipy* molecule upon complexation: the trans-arrangement of the pyridine rings in the free ligand is changed for the cis-arrangement in the complex.

In the IR spectra of complex **V**, the most intense bands correspond to the vibrations of crystallization water molecules (3450–3200 cm^–1^) and to the stretching vibrations of the V = O bonds (at 980 cm^–1^). In the IR spectrum of this complex, the ligand vibrations are primarily responsible for the band at a frequency of 1625 cm^–1^, which is associated with the stretching vibrations of the CH = N bonds of *salen* ligand (**see abbreviation in** **Table 3**).

The crystal structures of vanadium compounds are also determined by using single-crystal X-ray diffraction. The crystal data and refinement parameters for the structures of compounds are presented in the **Methods**. The crystal data for structures (CIFfiles) of complexes **I**, **II** and **V** have been additionally deposited with the Cambridge Structural Database.

The observed values of hypoglycemic activity for vanadium complexes reasonably correspond to those calculated on the basis of QSAR models (Table 4). It was interesting that predictive value of IC_50_ (FFAs) for anionic V(V) complex **I** was better than the predictive values of IC_50_ (FFAs) for all neutral V(IV) structures (see Table 4) contrary to the statement mentioned above.

**Table 4:** Predictive values of IC_50_ in mM (FFAs) for vanadium compounds and *in vivo* observations IC_50_ in mM (FFAs) and decreasing blood glucose level.

Compounds	VOSO_4_	Complex I	Complex II	Complex III	Complex IV	Complex V
***IC_50 predicted_***	–	0.50	0.75	0.53	0.93	0.87
***IC_50 exp_ (±S.D.)***	1.0±0.09	0.72±0.09	0.95±0.09	0.54±0.07	0.95±0.10	0.81±0.06
***decreasing blood glucose level***	16%	50%	33%	42%	11%	26%

*In vitro* study of the free fatty acids decrease amount released in adipocytes shows that FFAs level goes down in the presence of 0.5–1 mM of vanadium complexes (see Table 4) and all of them were more effective than VOSO_4_. The most effective complexes according to *in vitro* experiment were neutral complex **III** which belongs to VOL_2_ type and anionic complex **I** which belongs to [VO(O_2_)_2_L]^n-^ type vanadium compounds.

Recently it was shown that there is a correlation between glucose level and FFAs in normal and streptozotocin-diabetic rat (STZ-Rats) [20], [21]. It was observed that correlation coefficient of the liner regression (y = 177.5x+97.3) was 0.84 for a total of 64 for normal and STZ-Rats [20], [21]. This indicates that the serum FFAs level along with the serum glucose level is a good index of the degree of diabetes mellitus.

**Figure 6:**
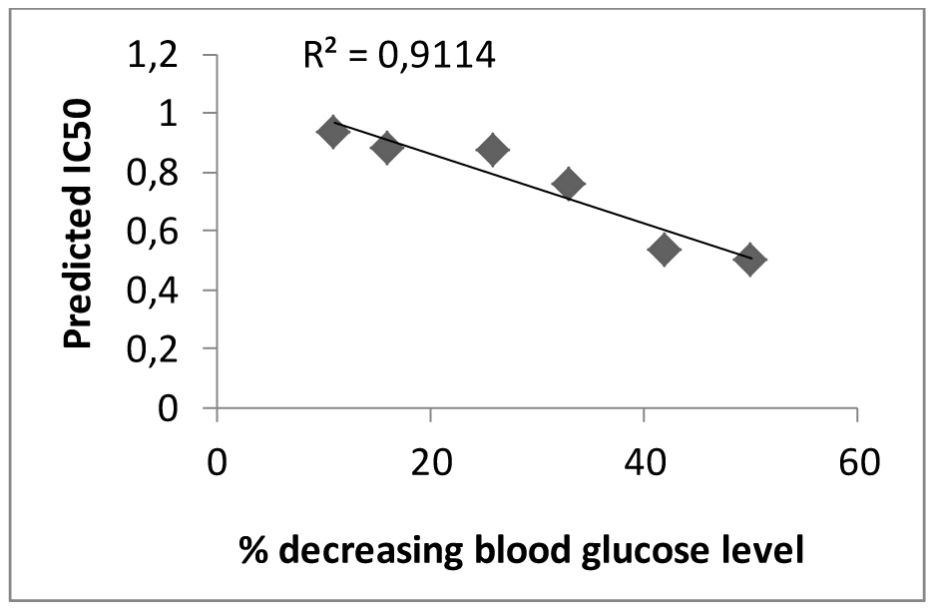
Correlation between experimental and calculated values for vanadium compounds.

In order to test whether the correlation between glucose level (experimental data) and FFAs (predicted values) we carried out *in vivo* study of hypoglycemic activity of our complexes **I–V**. *In vivo* hypoglycemic activity was determined in the model of adrenaline hyperglycemia caused by subcutaneous administration of 0.1% epinephrine hydrochloride. The results of decreasing blood glucose level (BGL) by vanadium compounds which were expressed in the percentage are summarized in Table 4. The good correlation (R^2^ = 0.91) between experimental and calculated values was observed (Fig. 6).

Estimation of rodent acute toxicity is an important task in drug design and risk assessment of chemicals. LD_50_ value is one of the important characteristics of acute toxicity that corresponds to the dose causing 50% mortality within 24 hours of administration. In order to evaluate the toxicity of selected complexes, we used “GUSAR online” web service which allows predicting the acute rat toxicity (LD_50_ values) with oral administration for compounds based on their structures. Obtained values of LD_50_ for selected complexes and experimental data are shown in Table 5. It should be noted that the class of toxicity and values of LD_50_ for all tested complexes were predicted accurately.

**Table 5:** Predicted and observed values LD_50_ of vanadium compounds.

#	Compounds	LD_50pred/_Class in AD	LD_50exp/_Class in AD
1	VOSO_4_	518.6/3	448(356–540)/3
2	Complex I	279.0/3	108(95–122)/3
3	Complex II	251.0/3	250(210–280)/3
4	Complex III	211.0/3	190(170–210)/3
5	Complex IV	357.1/3	261(210–312)/3
6	Complex V	265.1/3	245(210–280)/3

## Conclusions

Five coordination complexes of vanadium were synthesized and tested *in vivo* for their hypoglycemic activities and toxicity and were compared with the predictive data. Reasonable correspondence between the experimental and predicted values of toxicity and hypoglycemic activity for vanadium compounds indicates that GUSAR software may be successfully applied to explore the structure-activity relationships of metal complexes. Bis{tert-butyl[amino(imino)methyl]carbamato}oxovanadium(IV) which IC_50_ value was almost twice less than those obtained by vanadyl sulfate was identified as the most potent hypoglycemic agent among the synthesized compounds. We have shown that it may be possible to develop some vanadium compounds which have stronger selectivity against FFAs.

## Methods

### QSAR Method

GUSAR (version 2011) is based on Multilevel and Quantitative Neighborhoods of Atoms (MNA, QNA) descriptors [13], [14]. The calculation of QNA descriptors is based on the connectivity matrix (C), and also, on the standard values of ionization potential (IP) and electron affinity (EA) of atoms in a molecule [11], [12].

For any given atom *i*, the QNA descriptors are calculated as follows:
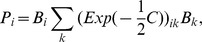


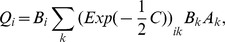



with 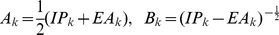.

The P and Q values are calculated for all atoms of the molecule. Two-dimensional Chebyshev polynomials are used for approximating the functions P and Q. Thus, the independent regression variables are calculated as average values of particular two-dimensional Chebyshev polynomials of P and Q values for the atoms in a molecule.

In addition, GUSAR allows the creation of QSAR models based on predicted biological activity profiles of compounds. This is done by using the PASS algorithm on each compound’s representation as a list of MNA descriptors for the prediction of the compound’s biological activity profile [22], [23]. The PASS program version of 10.1 predicts 4130 types of biological activity with a mean prediction accuracy of about 95%. The list of predicted biological activities includes pharmacotherapeutic effects, mechanisms of action, adverse and toxic effects, metabolic terms, susceptibility to transporter proteins and activities related to gene expression. The results of the PASS procedure are output as a list of the difference between the probability, for each biological activity, of the compound to be active (P_a_) or to be inactive (P_i_). For obtaining the different QSAR models in GUSAR, subsets of these P_a_-P_i_ values were randomly selected from the total list of predicted biological activities as input independent variables for the regression analysis. The size of these subsets depends on the number of compounds in the training set. If the number of compounds in the training set falls between 10 and 30, then the number of initial variables is closer to the number of compounds in the training set.

GUSAR (version 2011) also allows the calculation of whole-molecule descriptors: topological length, topological volume and lipophilicity. These descriptors are used in combination with the QNA and MNA descriptors.

For generation of the QSAR models, GUSAR uses a self-consistent regression (SCR) algorithm. SCR is based on the regularized least-squares method. Unlike stepwise regression and other methods of combinatorial search, the initial SCR model includes all regressors. The basic purpose of the SCR method is to remove the variables that poorly describe the modeled value but to retain the set of variables correctly representing the existing relationship. The number of final variables in the QSAR equation selected after the SCR procedure is typically significantly lower than the number of the initial variables. The details of the algorithms for descriptor calculation and the self-consistent regression methods have been described previously [22], [23].

For assessment of the applicability domain of the obtained models GUSAR uses the three different approaches: similarity, leverage, and accuracy assessment. The detailed description of these approaches is presented here [24].

The QSAR models obtained using both MNA and QNA descriptors are integrated into the consensus model which is further used for prediction. The final predicted value is estimated by taking into account a weighted average of the predicted values from each obtained QSAR model (QSAR models provide the predictions that are within the respective applicability domains). The predicted value obtained from each developed model is weighted on similarity value calculated during the evaluation of applicability domain.

### Chemistry Method

#### General methods for preparation of the vanadium derivatives

All reagents and solvents were used without further purification or drying. All the reagents (including reference material VOSO_4_) were purchased from Vekton (Russia). All reactions were proceeded in thermostat reactor under argon atmosphere. Beforehand, the solvents were dried and distilled under nitrogen using standard methods. Elementary analyses of H, C and N were performed with a Carlo Erba microanalyzer. ^1^H-NMR spectra at 400MHz were recorded on Varian XR-400 instrument, ^51^V-NMR spectra at 52.6MHz were recorded on Tesia Bruker AL-200 instrument, and mass spectra were obtained on a Finnigan MAT 112S spectrometer. IR spectra were obtained on a Specord M80 (Carl Zeiss) spectrophotometer within the spectral range of 400–4000 cm^−1^ under resolution of 2.5–4 cm^−1^. Spectra of the solutions in CH_2_Cl_2_ or CHCl_3_ were shown to represent pure solvents; the films were obtained on a KBr substrate by evaporation of the solvent. X-Ray diffraction study of the single crystal was performed on an Enraf-Nonius CAD4 automated diffractometer. The unit cell parameters were determined and refined using 25 reflections in the range of 15°≤θ≤16°(λ_MoKa_ radiation graphite monochromator).

#### Synthesis of compound I - Na[VO(O_2_)_2_(bipy)]·8H_2_O

Complex **I** was prepared following the procedure reported in [16]. A solution of 2,2′-pipydidine (0.8 g) in ethanol (10 ml) was added to a solution of sodium metavanadate in cooled 20% H_2_O_2_ (20 ml). After 2 to 3 min, ethanol (20 ml) was added with stirring and the solution was allowed to crystallize at 5°C. The yellow crystals thus formed were filtered off, washed once with ethanol, and dried. According to the data of elemental analysis, the resulting compound **I** had the composition Na[VO(O_2_)_2_(bipy)]·8H_2_O. The crystals are triclinic: *a* = 7.200(4) Å, *b* = 11.245(4) Å, *c* = 13.703(5) Å, α = 111.66(2)°, β = 90.86(2)°, γ = 88.01(2)°, *Z* = 2, *V* = 1030.5(8) Å, space group 1, ρ_calcd_ = 1.464 g/cm^3^, µ_Mo_  = 0.564 mm^−1^. ^1^H NMR, ^51^V NMR spectra, IR spectra and X-ray data of the synthesized compound **I** have been described in details early by Fedorova at al [25].

#### Synthesis of compound II - [VO(C_5_H_7_O_2_)_2_]

The oxovanadium complex [VO(*acac*)_2_] (**II**) was synthesized according to a modified procedure described in [17]. A mixture of V_2_O_5_ (20 g, 0.11 mol), distilled water (50 ml), concentrated sulfuric acid (30 ml), and ethanol (100 ml) was boiled with stirring for 30 min. The dark blue solution obtained was filtered, and freshly distilled acetylacetone (50 ml, 0.49 mol) was added to the filtrate. Then, a solution of sodium carbonate (80 g of Na_2_CO_3_ in 500 ml of water) in water (50 ml) was slowly added to the reaction mixture. The blue product was filtered off, washed with water, and dried in air. The yield was 50 g. Blue crystals were obtained by recrystallization from chloroform. The crystals were insoluble in water and soluble in CH_2_Cl_2_ and dimethyl sulfoxide. The crystal structure of compound **II** was determined using single-crystal X-ray diffraction. Crystals of compound **II** are triclinic, *a = *7.4997(19) Å, *b = *8.2015(15) Å, *c = *11.339(3) Å, α = 91.37(2) °, β = 110.36(2)°, γ = 113.33(2) °, *Z = *2, and space group *P*1.^ 1^H NMR, ^51^V NMR spectra, IR spectra and X-ray data of the synthesized compound **II** have been described in details early by Fedorova at al [26].

#### Synthesis of compound III - [VO(C_6_H_12_O_2_N_3_)_2_]

Complex **III** was prepared following the procedure reported in [18]. A solution of vanadyl sulfate VOSO_4⋅_2H_2_O in water was slowly added to the solution of tert-butyl carbamimidoylcarbamate in molar ratio 1∶2,2 with continuous stirring. Thereafter, the solution acquired a dark green color. In a matter of minutes, a heavy precipitate was formed. After stirring for 30 min, the substance was filtered off and washed with distilled water, 50% ethanol solution, and ethyl ether. The compound synthesized was dried in a vacuum desiccator at a temperature of 40°C. The yield of the product was approximately equal to 75% with respect to VOSO_4_⋅2H_2_O. The final product was recrystallized from methanol.

#### Synthesis of compound IV - K_4_[V_2_O_2_(C_4_H_4_O_6_)_2_]·2H_2_O

Complex **IV** was prepared following the procedure reported in [19]. A mixture of V_2_O_5_ distilled water and water solution of *D*-tartaric acid was boiled with stirring for 30 min. Then, a solution of potassium hydroxide in water was slowly added to the reaction mixture. The yield was 45%. The crystal structures of compound **IV** was determined using single-crystal X-ray diffraction. Complex **IV** with square–pyramidal coordination geometry, space group P4(3)22, *a* = 7.9345 Å, *b* = 7.9345 Å, *c* = 30.5035 Å, *V* = 1920.39(22) Å^3^, Z = 4, R_w_ = 3.11%. ^1^H NMR, ^51^V NMR spectra, IR spectra and X-ray data of the synthesized compound **IV** have been described in details earlier by Fedorova at al [27].

#### Synthesis of compound V - [VO(C_10_H_7_O_3_)(H_2_O)]

Oxovanadium complex **V** was synthesized according to a modified procedure described earlier in [17]. The ligand in the complex is a bivalent tridentate Schiff’s base, which was produced by the reaction of salicylaldehyde (*Sal*) with α-amino acid. *L*-alanine amino acid (0.1 mol) and sodium acetate (0.2 mol) were dissolved in distilled water (200 ml). In order to dissolve the amino acid completely, the solution was heated and filtered. A solution of salicylaldehyde (0.1 mol) in ethanol (250 ml) was added to the filtrate. A solution of vanadyl sulfate VOSO_4_⋅2H_2_O (0.085 mol) in water (80 ml) was slowly added to the above solution with continuous stirring. Thereafter, the solution acquired a dark brown color. In a matter of minutes, a heavy precipitate was formed. After stirring for 30 min, the substance was filtered off and washed with distilled water, 50% ethanol solution, and ethyl ether. The compound synthesized was dried in a vacuum desiccator at a temperature of 40°C. The yield of the product was approximately equal to 80% with respect to VOSO_4_⋅2H_2_O. The final product was recrystallized from metha similarity nol. The blue crystals thus prepared were insoluble in water, acetone, ether, and benzene and soluble in methanol, pyridine, methylene chloride, and chloroform. The melting temperature of the crystals was estimated as *T*_m_ ∼ 250°C. Crystals of compound **V** are monoclinic, *a = *8.5106(16) Å, *b = *7.373(2) Å, *c = *9.1941(16) Å, β = 101.88(1), *Z = *2, and space group *P*2_1_.^ 1^H NMR, ^51^V NMR spectra, IR spectra and X-ray data of the synthesized compound **V** have been described in details early by Fedorova at al [26].

### Pharmacology Method

All procedures were performed according to institutional guidelines for animal experimentation; protocol was submitted and approved by the Institutional Committee for Laboratory Animal Use and Care of the Saint-Petersburg Chemical Pharmaceutical Academy. Adult male Wistar rats and mongrel mice obtained from the ‘Rappolovo’ lab animals breeding farm near St. Petersburg, belonged to Russian Academy of Medical Science were used. All experiments were approved by the Animal Ethics Committee of the Saint-Petersburg Chemical Pharmaceutical Academy.

#### Insulin-mimetic action of vanadium complexes

All rats (250–300 g) and mice (22–25 g) were housed in a temperature controlled room (24°C±1°C) and they had received standard food pellets and tap water until they weighed. At the end of the study (24 h after CIN induction), all animal test subjects were euthanized by inhalation of ether anesthesia. Rat adipocytes were prepared from the fat pads of male Wistar rats by collagenase digestion according to the method of Rodbell [28]. Freshly prepared adipocytes were incubated (37*°*C, 30 min*)* with solutions of the vanadium compounds at three concentrations (0.1, 0.5 and 1 mM in isotonic saline and *5 *mM glucose), followed by 3 hours of incubation with epinephrine. The inhibitory effect was determined in terms of the decrease of the amount free fatty acids released in adipocytes. All assays were performed in duplicate or triplicate. **Table 4** summarizes the IC_50_ values, which are defined as the concentrations *c*(V) at which 50% inhibition of FFA release takes place. Additionally, five coordination complexes of vanadium and reference material VOSO_4_ have been tested *in vivo* for their hypoglycemic activities. Investigations were carried out on mongrel white mice.

#### Hyperglycemic activity

Determination of hypoglycemic activity of 5 complexes was performed on 75 mice. Adrenaline hyperglycemia was caused by subcutaneous administration of 0.1% epinephrine hydrochloride (AGH) at a dose of 1 mg/kg. With a single run all the compounds were administered orally 1 hour prior to the AGH, at 1/10 of the LD_50_. In each experimental group there were 3 animals and 3 replicates were performed. The content of glucose in whole blood was determined at 0.5, 1 and 2 hours after administration of AGH or glucose using portable glucose monitoring systems, OneTouch Ultra test strip and OneTouch Ultra and Clever check TD4209 and strips Clever check TD4209. Oral administration of all compounds elicited a progressive reduction in plasma glucose over 2 hours in mice. The results (as % decreasing blood glucose level) are summarized in **Table 4**.

#### Acute toxicity studies

Acute toxicity studies were carried out by rapid method of Prozorovskii [29]. The method consists of sequential administration of multiple doses with logarithmic step, resulting in a set number of responses and in determining the value of LD_50_, its range of variation of the average error using 4 groups of 3 observations in each group. The following criteria were used for the evaluation of acute toxicity: the number of dead animals, the timing of their death, LD_50_ through the night, the picture of intoxication, behavioral change and autopsy results of the survived animals after 14 days. Clinical examination was performed for each animal during the first hour after administration of the substance, and weekly thereafter. Clinical examination included a detailed inspection of the animal in cage, in hands and in the open area. The manifestation and severity of intoxication were noted. Examination of animals in order to identify mortality or signs of variations in health status was conducted on a daily basis. Integrated indicators were assessed weekly during the entire period of observation. Death of animals was recorded daily during entire study period. General condition and behavior of animals as well as type of toxic manifestations, including their development and termination were recorded daily during the procedures for monitoring and inspection of individual animals.

#### Statistical analysis

For all recorded indicators the following parameters of descriptive statistics were introduced: the arithmetic mean, the standard deviation and the standard error of the mean. The following statistical tools were used as methods for comparison: U- Mann-Whitney and Student’s tests; the Friedman and Wilcoxon test for repeated measurements; the U- Mann-Whitney for subsequent pairwise (post-hoc) comparisons. The level of comparison significance in this study was set up at 5%. Results were considered statistically significant if the p-value for the test was less than or equal to 0.05.

## Supporting Information

File S1 **List of twenty seven vanadium and zinc complexes with data on their hypoglycemic activity used for creating the QSAR models.**
(DOCX)
