# Supplementary material for: Design, Synthesis and Pharmacological Evaluation of Novel Vanadium-Containing Complexes as Antidiabetic Agents
Source: PLoS One. 2014 Jul 24;9(7):e100386. doi: 10.1371/journal.pone.0100386 (PMC4109918; doi:10.1371/journal.pone.0100386)
Supplement: File S1 — List of twenty seven vanadium and zinc complexes with data on their hypoglycemic activity used for creating the QSAR models. (DOCX) [file pone.0100386.s001.docx]

Supplement A

**List of twenty seven vanadium and zinc complexes with data on their hypoglycemic activity used for creating the QSAR models**

| **No.** | **Structural Formula** | **Code/Smiles** | **IC_50_, mmol/kg** | **Reference** |
| --- | --- | --- | --- | --- |
| 1 |  | VO(3hpa)2/[V+4]12(=O)([n+]3cccc(c3C(=O)O1)O)OC(=O)c1[n+]2cccc1O | 0.47 | 1 |
| 2 |  | bis(3-hydroxy-4-pyronato)oxovanadium(IV)/ O1C=C[C@@H]2C(=C1)O[V+4]1(=O)(O[C@@H]3C(=COC=C3)O1)O2 | 1.179 | 2 |
| 3 |  | VO(3mpa)2/[V+4]12(=O)([n+]3c(C(=O)O2)c(ccc3)C)[n+]2c(C(=O)O1)c(ccc2)C | 0.66 | 3 |
| 4 |  | VO(4clpa)2/[V+4]12(=O)([n+]3c(C(=O)O2)cc(cc3)Cl)[n+]2c(C(=O)O1)cc(cc2)Cl | 0.7 | 3 |
| 5 |  | VO(5ipa)2/[V+4]12(=O)([n+]3c(C(=O)O2)ccc(c3)I)[n+]2c(C(=O)O1)ccc(c2)I | 0.45 | 3 |
| 6 |  | VO(6epa)2/[V+4]12(=O)([n+]3c(C(=O)O2)cccc3CC)[n+]2c(C(=O)O1)cccc2CC | 0.81 | 4 |
| 7 |  | VO(6hpa)2/[V+4]12(=O)([n+]3c(C(=O)O2)cccc3O)[n+]2c(C(=O)O1)cccc2O | 0.8 | 1 |
| 8 |  | VO(6mpa)2/[V+4]12(=O)([n+]3c(C(=O)O2)cccc3C)[n+]2c(C(=O)O1)cccc2C | 0.49 | 3 |
| 9 |  | VO(alx)2/[V+4]12(=O)([O+]=C3C(=C(OC(=C3OC)C)CCCCC)O1)[O+]=C1C(=C(OC(=C1OC)C)CCCCC)O2 | 0.553 | 5 |
| 10 |  | bis(ethylmaltolato)oxovanadium(IV)/ [V+4]12(=O)(O[C@@H]3C(=C(OC=C3)CC)O1)O[C@@H]1C(=C(OC=C1)CC)O2 | 0.632 | 5 |
| 11 |  | bis(kojiato)oxovanadium(IV)/ [V+4]12(=O)(O[C@@H]3C(=COC(=C3)O)O1)O[C@@H]1C(=COC(=C1)O)O2 | 2.37 | 5 |
| 12 |  | bis(maltolato)oxovanadium(IV)/ [V+4]12(=O)(O[C@@H]3C(=C(OC=C3)C)O1)O[C@@H]1C(=C(OC=C1)C)O2 | 0.676 | 5 |
| 13 |  | VO(opt)2/[V+4]12(=O)(Sc3[n+](O2)cccc3)Sc2[n+](O1)cccc2 | 0.19 | 6 |
| 14 |  | VO(pic)2/[V+4]12(=O)([n+]3c(C(=O)O2)cccc3)[n+]2c(C(=O)O1)cccc2 | 0.59 | 3 |
| 15 |  | Zn(6mpa)2/[Zn@@+2]12([n+]3c(C(=O)O2)cccc3C)[n+]2c(C(=O)O1)cccc2C | 0.31 | 7 |
| 16 |  | Zn(maltolat)2/[Zn+2](Oc1c(=O)ccoc1C)Oc1c(=O)ccoc1C | 0.59 | 7 |
| 17 |  | Zn(L-Thr)2/[Zn@+2]12(OC(=O)[C@H]([NH2+]2)[C@H](O)C)OC(=O)[C@H]([NH2+]1)[C@H](O)C | 0.54 | 8 |
| 18 |  | Zn(pic)2/[Zn@+2]12([n+]3c(C(=O)O2)cccc3)[n+]2c(C(=O)O1)cccc2 | 0.64 | 7 |
| 19 |  | Zn(L-Pro)2/[Zn+2](N1[C@@H](C=O)CCC1)N1[C@@H](C=O)CCC1 | 0.89 | 8 |
| 20 |  | ZnSO4/ S1(=O)(=O)O[Zn+2]O1 | 0.81 | 8 |
| 21 |  | Zn(L-Val)2/[Zn+2](N[C@@H](C=O)C(C)C)N[C@H](C=O)C(C)C | 0.77 | 8 |
| 22 |  | Zn(L-Asn)2/[Zn@@+2]12(NC(=O)C[C@@H](N1)C=O)NC(=O)C[C@H](N2)C=O | 0.65 | 8 |
| 23 |  | [VIVO(bp-O)HSO4]/ [V+4]123(=O)([N@+](Cc4[n+]1cccc4)(Cc1[n+]2cccc1)Cc1c(O3)cccc1)OS(=O)(=O)O | 3.85 | 9 |
| 24 |  | [VIVO(tpa)SO4]/ V+4]123(=O)[N+](Cc4[n+]1cccc4)(Cc1[n+]3cccc1)Cc1[n+]2cccc1 | 4.6 | 9 |
| 25 |  | [VVO(pic-trpH)tpa]2+/[V+4]12(=O)([n+]3c(C(=O)O1)ccc(c3)C(=O)N[C@@H](C(=O)O)Cc1c[nH]c3c1cccc3)[N@@+](Cc1[n+]2cccc1)(Cc1ncccc1)Cc1ncccc1 | 1.84 | 9 |
| 26 |  | [VIVO(pic-trpMe)2]/ [V+4]12(=O)([n+]3c(C(=O)O2)ccc(c3)C(=O)N[C@@H](C(=O)OC)Cc2c[nH]c3c2cccc3)[n+]2c(C(=O)O1)ccc(c2)C(=O)N[C@@H](C(=O)OC)Cc1c[nH]c2c1cccc2 | 0.41 | 9 |
| 27 |  | Vanadyl sulfate/[V+4]1(=O)OS(=O)(=O)O1 | 0.84 | 1 |

**References**

1. Hiromu Sakurai, Hiroyuki Yasui (2003) Structure-activity relationship of insulinomimetic vanadyl-picolinate complexes in view of their clinical use: *The Journal of Trace Elements in Experimental Medicine: 16: 269–280. DOI: 10.1002/jtra.10036*
2. Hiromu Sakurai, Yutaka Yoshikawab and Hiroyuki Yasuib (2008) Current state for the development of metallopharmaceutics and anti-diabetic metal complexes: *Chem. Soc. Rev:* 37: 2383-2392. DOI: 10.1039/B710347F
3. Sakurai H, Tamura A, Takino T, Ozutsumi K, Kawabe K, Kojima Y. (2000) Interaction of vanadyl complexes with biological systems: structure-insulinomimetic activity relationship of vanadyl_picolinate complexes. *Inorg React Mech:* 2: 69-77.
4. Sasagawa T, Yoshikawa Y, Kawabe K, Sakurai H, Kojima Y. (2002) Bis(6-ethylpicolinato) oxovanadium(IV) complex with normoglycemic activity in KK-Ay mice. *J Inorg Biochem*; 88: 108-112.
5. Hiromu Sakurai, Akira Katoh, Tamas Kiss, Tamas Jakusch and Masakazu Hattori (2010) Metallo–allixinate complexes with anti-diabetic and anti-metabolic syndrome activities. *Metallomics: 2: 670-682. DOI: 10.1039/c0mt00025f*
6. Hiromu Sakurai, Hiromi Sano, Toshikazu Takino and Hiroyuki Yasui (1999) A new type of orally active insulin-mimetic vanadyl complex: bis(1-oxy-2-pyridinethiolato)oxovanadium(IV) with VO(S2O2) coordination mode: *Chemistry Letters*: 28: 913-914. DOI:10.1246/cl.1999.913
7. Yoshikawa Y, Ueda E, Kawabe K, Miyake H, Takino T, Sakurai H and Kojima Y. (2002) Development of new insulinomimetic zinc(II) picolinate complexes with a Zn(N2O2) coordination mode: structure characterization, in vitro, and in vivo studies. *J Biol Inorg Chem*: 7(1-2): 68-73.
8. Yutaka Yoshikawa, Eriko Ueda, Yuka Sukuzi, Naohisa Yanagihara, Hiromu Sakurai, and Yoshitane Kojima (2001) New Insulinomimetic Zinc(II) Complexes of a-Amino Acids and Their Derivatives with Zn(N2O2) Coordination Mode: *Chem. Pharm. Bull.* 49(5): 652—654.
9. Jessica Nilsson, Eva Degerman, Matti Haukka, George C. Lisensky, Eugenio Garribba, Yutaka Yoshikawa, Hiromu Sakurai, Eva A. Enyedy, Tamás Kiss, Hossein Esbak, Dieter Rehder and Ebbe Nordlander (2009) Bis- and tris(pyridyl)amine-oxidovanadium complexes: Characteristics and insulin-mimetic potential. *Dalton Trans.38: 7902-7911. 10.1039/B903456K*
